# The Role of 1-3-Beta-D-Glucan in Predicting *Pneumocystis jirovecii* Pneumonia in Immunocompromised Children

**DOI:** 10.1093/jpids/piag085

**Published:** 2026-08-29

**Authors:** Markus Keldorfer, Raphael Ulreich, Johann Martensen, Patrick Faudon, Klaus Pfurtscheller, Siegfried Rödl, Robert Krause, Karl Dichtl, Volker Strenger, Stefan Hatzl

**Affiliations:** Pediatric Intensive Care Unit, Department of Children and Adolescent Medicine, Medical University of Graz, Graz, Austria; Pediatric Intensive Care Unit, Department of Children and Adolescent Medicine, Medical University of Graz, Graz, Austria; Pediatric Intensive Care Unit, Department of Children and Adolescent Medicine, Medical University of Graz, Graz, Austria; Pediatric Intensive Care Unit, Department of Children and Adolescent Medicine, Medical University of Graz, Graz, Austria; Pediatric Intensive Care Unit, Department of Children and Adolescent Medicine, Medical University of Graz, Graz, Austria; Pediatric Intensive Care Unit, Department of Children and Adolescent Medicine, Medical University of Graz, Graz, Austria; Division of Infectious Diseases, Department of Internal Medicine, Medical University of Graz, Graz, Austria; Diagnostic and Research Institute of Hygiene, Microbiology and Environmental Medicine, Medical University of Graz, Graz, Austria; Division of General Pediatrics, Department of Children and Adolescent Medicine, Medical University of Graz, Graz, Austria; Research Unit for Host-Pathogen Interaction, Medical University of Graz, Graz, Austria; Intensive Care Unit, Department of Internal Medicine, Medical University of Graz, Graz, Austria

**Keywords:** *Pneumocystis jirovecii* pneumonia, 1,3-β-D-glucan, pediatric, immunocompromised, non-HIV, diagnostic accuracy

## Abstract

Serum 1,3-β-D-glucan (BDG) showed high negative predictive value (~99%) for *Pneumocystis jirovecii* pneumonia (PJP) in 106 non-human immunodeficiency virus immunocompromised children, but modest positive predictive value at low prevalence. These exploratory findings suggest BDG may help exclude PJP, pending prospective validation.

## INTRODUCTION

*Pneumocystis jirovecii* pneumonia (PJP) causes significant mortality and morbidity in immunocompromised children, yet diagnostic evidence in this population is almost entirely extrapolated from adult data. In recent a meta-analysis evaluating prophylactic and therapeutic regimens, fewer than 2% of included patients were children, and none were toddlers or infants. ^1^ The epidemiology of PJP has shifted toward non-human immunodeficiency virus (HIV) immunocompromised populations, including children with hematologic malignancies, solid tumors, and stem cell transplant recipients, a group in which pediatric patients remain similarly underrepresented in the literature.^1^^,^^2^ Early and accurate diagnosis is essential, as timely treatment significantly improves outcomes. Bronchoalveolar lavage (BAL) provides the highest diagnostic sensitivity but may carry procedural risk in critically ill children, making non-invasive adjunct tests valuable. Serum 1,3-β-D-glucan (BDG) has emerged as one such adjunct, but major systematic reviews of its diagnostic accuracy provide no pediatric subgroup analyses and extrapolation from adult data is common.^3^ We therefore aimed to evaluate the diagnostic performance of BDG and clinical predictors of PJP in a pediatric cohort, contributing data to this underserved area.

## METHODS

We conducted a single-center retrospective observational study including all consecutive immunocompromised children who underwent testing for *Pneumocystis jirovecii* in respiratory samples and fulfilled revised European Organization for Research and Treatment of Cancer (EORTC) host-factor criteria.^4^  *P. jirovecii* polymerase chain reaction (PCR) has been available at our institution since 2013, with no transition from microscopy to PCR during the study period; the earliest test captured in the cohort dates from 2015, and the study period spans 2015 to March 2023. The reference standard for PJP was a positive *P. jirovecii*–specific PCR performed on BAL; serum BDG was not used in case ascertainment, thereby avoiding incorporation bias. Cases were classified as proven, probable, or no PJP according to the 2021 European Organization for Research and Treatment of Cancer and the Mycoses Study Group Education and Research Consortium (EORTC/MSGERC) criteria^4^^,^^5^: Proven PJP required microscopic or histologic visualization of the organism (established at post-mortem examination in the patients who died), and probable PJP required a positive BAL PCR with compatible host factors and clinical-radiologic features. To distinguish true PJP from colonization, all PCR-positive cases were independently reviewed by an infectious disease specialist and a pediatrician; cases with an alternative primary infectious etiology, clinical improvement without anti-*Pneumocystis* therapy, and high PCR cycle-threshold (Ct) values were classified as colonization by consensus and excluded. Confirmed PJP cases had a median Ct of 25 (range 22–31) versus 39 (range 37–42) in colonization cases.

Serum BDG was measured using the Fungitell assay (Associates of Cape Cod), as previously described.^3^ Laboratory, clinical, and radiology data were extracted from our institutional electronic database using REDCap.^6^ Two covariates with missing data (prior stem cell transplantation status, *n* = 167; concurrent viral detection, *n* = 168) were imputed using multiple imputation by chained equations (MICE; *m* = 20, predictive mean matching). All laboratory values were complete and were not imputed. The index test (serum BDG) and the reference standard (PCR) were complete for all evaluable observations and were never imputed; all 171 evaluable tests had a concurrent measured BDG underpinning the receiver operating characteristic analysis. Firth penalized logistic regression was applied due to low event rates, with 4 predictors pre-specified prior to analysis based on clinical rationale: BDG (per 100 pg/mL), lactate dehydrogenase (LDH, per 100 U/L), PJP prophylaxis status, and bilateral pulmonary infiltrates on chest imaging.^7^^,^^8^ To address within-patient correlation from repeated testing, a patient-level analysis restricted to the index (earliest evaluable) test per patient was pre-specified and is reported as co-primary alongside the test-level analysis. Diagnostic accuracy of serum BDG was evaluated at both the test and patient level by receiver operating characteristic (ROC) analysis with bootstrap internal validation (1000 resamples; Harrell optimism correction); predictive values were calculated at the data-derived (33 pg/mL) and manufacturer-recommended (≥80 pg/mL) cut-offs with exact (Wilson) 95% CIs. Absolute lymphocyte count (ALC) was added to the descriptive and univariable analyses as a recognized risk factor for PJP. A pre-specified sensitivity analysis reclassified the colonization cases as PJP. Institutional PJP prevalence was estimated using a Bayesian Beta-Binomial framework (Figure S1).^3^ The study was approved by the local institutional review board (EK Nr: 1207/2024) and conducted in accordance with the Declaration of Helsinki. Informed consent was waived given the retrospective design. A *P* value <0.05 was considered statistically significant.

## RESULTS

Among 171 tests from 106 unique pediatric patients without HIV infection (median age 7.6 years; IQR 2.6–12.9), 17 PCR tests (9.9%) were initially positive. Four were reclassified as colonization on consensus review, each having an alternative primary etiology (two SARS-CoV-2, one influenza A, one lobar pneumonia), clinical improvement without anti-*Pneumocystis* therapy, and a high cycle-threshold value (median Ct 39 vs 25 in confirmed cases), leaving 13 PJP-positive tests (7.6%) from 10 patients (9.4%); 3 patients underwent serial testing in a single episode. No additional fungal infections were detected across the cohort, and none of the 10 patients with PJP had a proven or probable invasive fungal infection in the 30 days before the PJP diagnosis. The most common underlying conditions were hematologic malignancy (47%), solid malignancy (14%), and prior stem cell transplantation (9.7%). PJP prophylaxis was used in 46% of patients; only 3 PJP-diagnosed patients had received prophylaxis. All children having PJP were treated with trimethoprim–sulfamethoxazole (TMP–SMX) at 15–20 mg/kg daily. Patient characteristics are summarized in Table 1.

**Table 1:** Baseline Characteristics of Immunocompromised Children Tested for *Pneumocystis jirovecii*

Characteristic	Overall N = 106 ^a^	PJP N = 10	No PJP N = 96 ^a^	*P-*value
**Age at test (years)**	7.6 (2.6-12.9)	2.3 (0.3-10.7)	8.0 (3.0-13.1)	0.061
**Sex**				0.5
Male	63 (59%)	5 (50%)	58 (60%)	
Female	43 (41%)	5 (50%)	38 (40%)	
**Pneumocystis diagnosis**				<0.001
BAL-PCR	49 (46%)	10 (100%)	39 (40%)	
Nasopharyngeal Swab PCR	57 (54%)	0 (0%)	57 (60%)	
**Primary immunosuppression category**				>0.9
Hematologic malignancy	50 (47%)	5 (50%)	45 (47%)	
HIV	0 (0%)	0 (0%)	0 (0%)	
Nephrologic disease	2 (1.9%)	0 (0%)	2 (2.1%)	
Other / unknown	33 (31%)	4 (40%)	29 (30%)	
Rheumatologic disease	3 (2.8%)	0 (0%)	3 (3.1%)	
Solid malignancy	15 (14%)	1 (10%)	14 (15%)	
Solid organ transplant	3 (2.8%)	0 (0%)	3 (3.1%)	
**Prior stem cell transplant (SCT)**	10 (9.7%)	0 (0%)	10 (11%)	0.6
**SCT type**				>0.9
alloSCT	8 (80%)	0 (0%)	8 (80%)	
autoSCT	2 (20%)	0 (0%)	2 (20%)	
**Solid organ transplant**	3 (2.9%)	0 (0%)	3 (3.3%)	>0.9
HTX	1 (33%)	0 (0%)	1 (33%)	
NTX	2 (67%)	0 (0%)	2 (67%)	
**Systemic glucocorticoids**	22 (21%)	2 (20%)	20 (21%)	>0.9
**PJP prophylaxis (≥ 7 days)**	49 (46%)	3 (30%)	46 (48%)	0.3
**Prophylaxis agent**				0.5
TMP-SMX	30 (63%)	1 (33%)	29 (64%)	
Pentamidine	18 (38%)	2 (67%)	16 (36%)	
**Rituximab therapy**	2 (1.9%)	0 (0%)	2 (2.1%)	>0.9
**ICU admission at time of test**	20 (19%)	2 (20%)	18 (19%)	>0.9
**Maximum ventilation support**				0.041
O2 supplementation	87 (82%)	8 (80%)	79 (82%)	
HFNC	4 (3.8%)	0 (0%)	4 (4.2%)	
NIV	2 (1.9%)	2 (20%)	0 (0%)	
Tracheostomy CPAP	1 (0.9%)	0 (0%)	1 (1.0%)	
Invasive ventilation	11 (10%)	0 (0%)	11 (11%)	
ECMO	1 (0.9%)	0 (0%)	1 (1.0%)	
**Leukocytes (G/L)**	6.0 (2.1-10.1)	6.1 (4.4-8.4)	6.0 (1.9-10.3)	0.6
**Absolute neutrophil count (G/L)**	3.8 (2.0-6.7)	4.1 (2.8-4.9)	3.7 (1.6-7.6)	>0.9
**Absolute lymphocyte count (G/L)**	0.7 (0.5-1.1)	0.2 (0.1–0.4)	0.8 (0.6–1.1)	<0.001
**Hemoglobin (g/dL)**	10.8 (9.3-12.6)	10.1 (9.2-11.9)	10.9 (9.3-12.8)	0.4
**Platelets (G/L)**	206.5 (86.0-332.0)	288.0 (100.0-352.0)	195.5 (79.5-323.0)	0.5
**CRP (mg/L)**	19.3 (3.2-61.7)	26.1 (1.5-128.3)	19.1 (3.5-57.0)	0.7
**LDH (U/L)**	234.5 (178.0-359.0)	489.0 (430.0-648.0)	223.5 (177.0-337.0)	<0.001
**Bilirubin (mg/dL)**	0.4 (0.3-0.6)	0.5 (0.4-0.5)	0.4 (0.2-0.6)	0.5
**Creatinine (mg/dL)**	0.4 (0.3-0.6)	0.3 (0.3-0.5)	0.4 (0.3-0.6)	0.4
**1,3-β-D-glucan (BDG) (pg/mL)**	16.0 (16.0-16.0)	542.5 (202.3-745.0)	16.0 (16.0-16.0)	<0.001
**Bilateral pulmonary infiltrates**	16 (15%)	4 (40%)	12 (13%)	0.042
**ICU admission within 14 days of positive PCR**	1 (13%)	1 (13%)	-	>0.9
**Died during follow-up**	14 (13%)	2 (20%)	12 (13%)	0.6

Characteristics are presented for all 106 patients overall and stratified by PJP status (*children with PJP, n* = 10; *children without PJP, n* = 96). Continuous variables are reported as median (interquartile range); categorical variables as *n* (%). *P*-values were calculated using the Wilcoxon rank-sum test for continuous variables and Fisher's exact test for categorical variables. Prior stem cell transplantation status (4 missing) and concurrent viral detection (3 missing) were imputed using multiple imputation by chained equations (MICE; *m* = 20, predictive mean matching). All laboratory values, including serum 1,3-β-D-glucan, and PCR results were complete (0% missing) and were not imputed. All PCR-positive patients received trimethoprim-sulfamethoxazole therapy, confirmed by chart review.

Abbreviations: BAL, bronchoalveolar lavage; BDG, 1,3-β-D-glucan; CRP, C-reactive protein; ECMO, extracorporeal membrane oxygenation; HFNC, high-flow nasal cannula; HTX, heart transplantation; ICU, intensive care unit; IQR, interquartile range; LDH, lactate dehydrogenase; NIV, non-invasive ventilation; NTX, renal transplantation; PJP, *Pneumocystis jirovecii* pneumonia; PCR, polymerase chain reaction; SCT, stem cell transplantation; TMP-SMX, trimethoprim-sulfamethoxazole.

For diagnosis, only *Pneumocystis jirovecii*-specific PCR was used, performed on BAL in 49 patients (46%) and nasopharyngeal swab (NPS) in 57 patients (54%). Importantly, all children with confirmed PJP underwent BAL (100%), while NPS without subsequent BAL was used exclusively in non-PJP patients (60%; *P* < 0.001).

**Figure 1:**
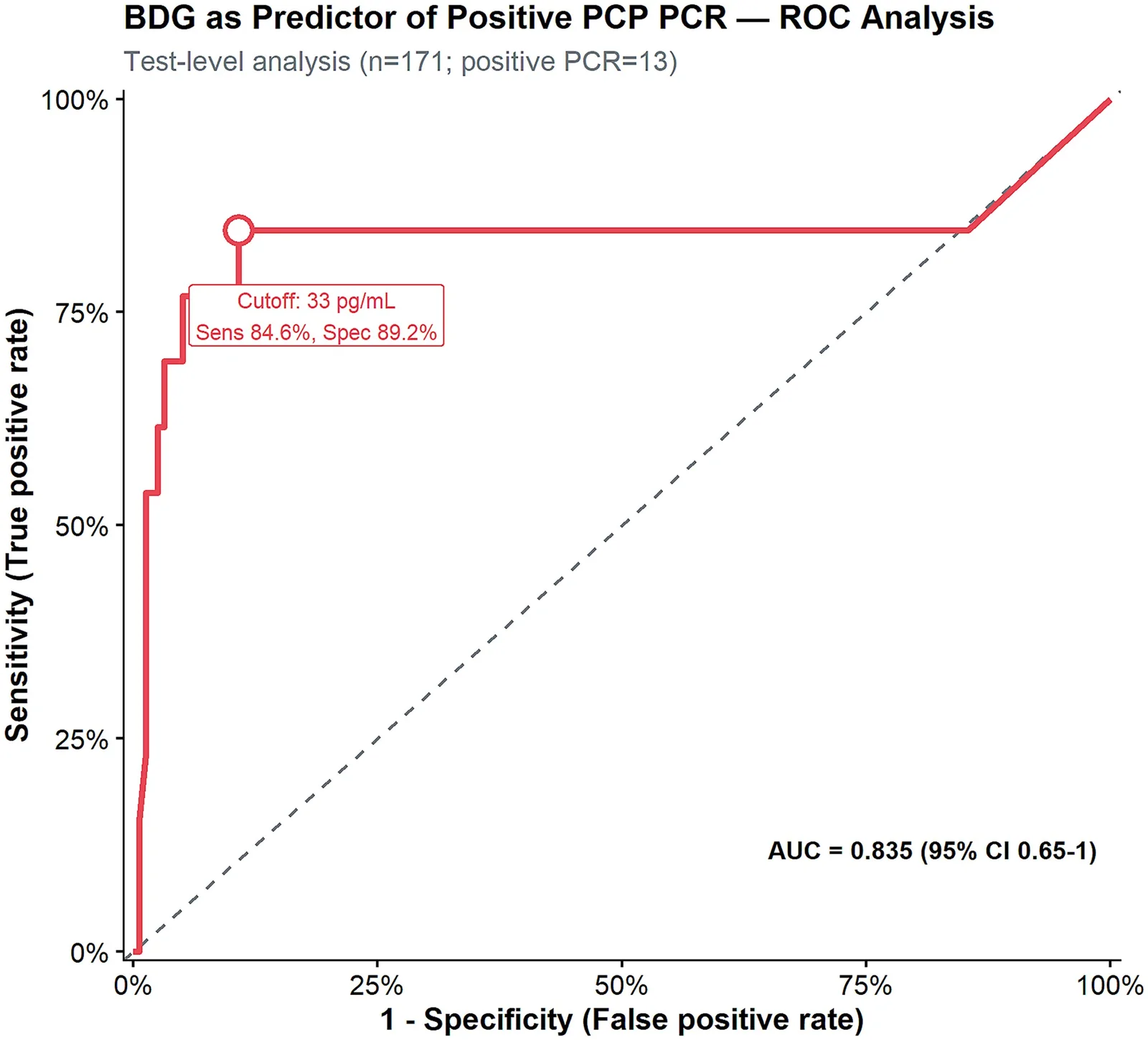
Receiver Operating Characteristic (ROC) Curve for Serum 1,3-β-D-Glucan as a Predictor of *Pneumocystis jirovecii* Pneumonia. ROC Analysis Performed at the Test Level (n = 171; Positive PCR = 13). The Youden-Optimal Cut-off of 33 Pg/mL Yielded a Sensitivity of 84.6% and Specificity of 89.2%, with an AUC of 0.835 (95% CI 0.65–1.00). Bootstrap Internal Validation (1000 Resamples; Harrell Optimism Correction) Produced a Bias-Corrected AUC of 0.837, Indicating Minimal Overfitting. The Wide Confidence Interval Reflects Imprecision Arising from the Small Number of Positive Cases. AUC, Area under the Curve; BDG, 1,3-β-D-Glucan; PJP, *Pneumocystis jirovecii* Pneumonia; PCR, Polymerase Chain Reaction

Serum BDG discriminated PJP from other respiratory disease, with a test-level AUC of 0.835 (95% CI, 0.65-1.00; Figure 1) and a concordant patient-level AUC of 0.829 (95% CI, 0.64-0.99). Bootstrap internal validation showed minimal optimism (−0.002; bias-corrected AUC 0.837), indicating the point estimate is not inflated by overfitting; however, the wide confidence interval reflects imprecision inherent to the small number of positive cases and limits the strength of conclusions. At the exploratory Youden-optimal threshold of 33 pg/mL, sensitivity was 84.6% and specificity 89.2%, corresponding to a positive predictive value (PPV) of 39.3% (95% CI, 23.6-57.6) and a high negative predictive value (NPV) of 98.6% (95% CI, 95.0-99.6). At the manufacturer-recommended ≥80 pg/mL cut-off, sensitivity was unchanged (84.6%) while specificity rose to 96.8%, PPV increased to 68.8% (95% CI, 44.4-85.8), and NPV remained high at 98.7% (95% CI, 95.4-99.6); patient-level estimates were concordant (sensitivity 90.0%, specificity 96.9%, NPV 98.9% at ≥80 pg/mL; Table S3). Notably, all 5 non-PJP tests with BDG ≥80 pg/mL had a documented exogenous source of glucan (immunoglobulin or albumin infusion, or cellulose-containing materials), indicating that the few false positives at the manufacturer cut-off reflect recognized exogenous sources rather than assay non-specificity. Among the four pre-specified predictors, BDG and LDH were independently associated with PJP in multivariable analysis: BDG (adjusted OR 1.37 per 100 pg/mL, 95% CI, 1.14-1.73; *P* = 0.001) and LDH (adjusted OR 1.51 per 100 U/L, 95% CI, 1.09-2.10; *P* = 0.014) (Figure S2). In the per-patient sensitivity analysis, BDG remained the dominant predictor (OR 1.84, 95% CI, 1.34-3.42; *p* < 0.001), while LDH lost significance (OR 1.17; *P* = 0.545), suggesting its primary association may reflect within-patient trends rather than an independent signal (Table S1). PJP prophylaxis and bilateral infiltrates did not reach statistical significance, though bilateral infiltrates were more prevalent among children having PJP in descriptive analysis (40% vs 13%; *P* = 0.042).

In a pre-specified sensitivity analysis reclassifying the 4 colonization cases as PJP, discrimination was materially unchanged (AUC 0.801, 95% CI, 0.61-0.97; BDG adjusted OR 1.42 per 100 pg/mL, 95% CI, 1.16-1.75).

In exploratory univariable analysis, C-reactive protein (OR 2.35 per 100 mg/L, 95% CI, 1.36-4.41; *P* = .003) and low ALC; (OR 0.15 per 1 G/L, 95% CI, 0.03-0.35; *P* < 0.001) were associated with PJP. Other hematologic parameters (leukocytes, absolute neutrophil count, hemoglobin, platelets) were not, indicating that general cytopenia does not discriminate PJP, whereas lymphopenia specifically does: children with PJP had a markedly lower ALC (median 0.2 vs. 0.8 G/L; *P* < 0.001), a difference evident despite their younger age, in whom higher lymphocyte counts would be expected (Table S2).

Overall survival was 97%, 96%, and 92% at 90, 180, and 365 days respectively. Among PJP patients, survival was 100% at 90 and 180 days, declining to 90% at one year, with two deaths over the observation period (Figure S3, Figure S4).

## DISCUSSION

In this single-center cohort of non-HIV immunocompromised children, serum BDG discriminated PJP with an AUC of 0.835, with point estimates broadly consistent with, though not confirmatory of, adult data. A meta-analysis of 26 studies using the Fungitell BDG assay reported a pooled sensitivity of 83.5% and specificity of 75.5% at the 80 pg/mL cut-off^3^; our pediatric estimates at the same cut-off (sensitivity 84.6%, specificity 96.8%) are concordant with this pooled sensitivity, though larger studies are required to establish comparability with confidence.

The Youden-optimal threshold of 33 pg/mL is substantially lower than adult cut-offs of 80 or 400 pg/mL and must be considered hypothesis-generating only. We do not propose a biologically distinct pediatric threshold: BDG reflects fungal burden and is not expected to differ by host age. The median BDG in uninfected children was 16 pg/mL, at the assay floor, so 33 pg/mL primarily separates any measurable signal from background rather than representing a biologically derived threshold. The lower background more plausibly reflects fewer iatrogenic sources of BDG in children (immunoglobulin and albumin infusions, certain antibiotics, cellulose-containing materials) than pediatric physiology.^9^^,^^10^ Consistent with this, all non-PJP tests reaching ≥80 pg/mL had a documented exogenous source. We therefore report performance at the standard ≥80 pg/mL cut-off, at which BDG functioned as a rule-out test (NPV ~99%), and note that external validation in larger pediatric cohorts is needed before any cut-off can be applied clinically.^11^

LDH should be considered a supportive rather than primary biomarker, given its attenuation in per-patient sensitivity analysis. Younger age emerged as a clinically notable signal, median age was 2.3 years in PJP patients versus 8.0 years in those without, and consistent with observations that infants and toddlers face heightened risk due to immune immaturity and prophylaxis challenges.^2^ Profound lymphopenia was the strongest univariable hematologic signal (median ALC 0.2 vs 0.8 G/L; OR 0.15 per 1 G/L) and is an established risk factor for PJP. That it persisted despite the younger age of cases, in whom lymphocyte counts are physiologically higher, supports it as a genuine risk marker rather than an age artifact. Three PJP cases occurred despite prophylaxis, two on inhaled pentamidine and one on TMP–SMX, reinforcing that a prophylaxis history should not lower clinical suspicion and that TMP–SMX remains the preferred agent when tolerated.

This study has several limitations. The small number of PJP events, 10 at the patient level, results in wide CIs, limits regression stability, and renders the BDG threshold hypothesis-generating only. The study is single-center and retrospective, limiting generalizability. BDG was measured on a single commercial assay and results may not transfer to other platforms. The absence of children living with HIV reflects current epidemiology in high-income settings but limits comparison with the adult literature. Importantly, respiratory sample heterogeneity does not compromise the reference standard: all PJP-confirmed patients underwent BAL, while NPS was used exclusively in non-PJP patients (*P* < 0.001).

In conclusion, serum BDG showed moderate but imprecise discrimination for PJP in non-HIV immunocompromised children and performed best as a rule-out test, reflecting its high negative predictive value at low prevalence. BDG was the most robust predictor across test- and patient-level analyses, whereas the LDH association was less consistent. These findings highlight the need for prospective, multicenter pediatric studies to establish and externally validate diagnostic thresholds for BDG.

## Supplementary Material

Supplementary_JPIDS_final_revised_piag085

## Data Availability

The anonymized dataset and full analysis code are available from the corresponding author upon reasonable request.
