# Supplementary material for: The Role of 1-3-Beta-D-Glucan in Predicting Pneumocystis jirovecii Pneumonia in Immunocompromised Children
Source: J Pediatric Infect Dis Soc. 2026 Aug 29;15(9):piag085. doi: 10.1093/jpids/piag085 (PMC13573584; doi:10.1093/jpids/piag085)
Supplement: Supplementary file 1 [file supplementary_jpids_final_revised_piag085.docx]

**Supplementary Figure 1: Bayesian Beta-Binomial Estimation of the Institutional PJP Rate in Immunocompromised Children**.

Posterior probability distributions for the true institutional rate of *Pneumocystis jirovecii* pneumonia (PJP) (θ) estimated using a Beta-Binomial conjugate model. The observed data comprised 13 positive results among 171 evaluable tests (7.6%), represented by the vertical dotted black line. Dashed lines show the three prior distributions specified before observing the data; solid lines show the corresponding posterior distributions after updating each prior with the observed likelihood.

Three priors were evaluated reflecting different assumptions about the expected positivity rate. The meta-analysis informed prior (purple; Beta(2.25, 7.75); prior mean 22.5%) was derived from the pooled PJP prevalence reported by Prosty et al. in a systematic review and meta-analysis of the Fungitell 1,3-β-D-glucan assay across 26 studies of immunocompromised adults (1150/5111 patients), discounted to an effective sample size of 10 to account for uncertainty in extrapolating adult data to a pediatric population. The pediatric-adjusted conservative prior (green; Beta(1.5, 8.5); prior mean 15%) assumed lower positivity rates reflecting stricter prophylaxis practices in children. The uninformative reference prior (orange; Beta(1,1)) placed equal probability on all values of θ from 0% to 100%, allowing the observed data to drive the posterior entirely.

Despite the three priors spanning a wide range of prior beliefs (15–22.5% prior means), all posterior distributions converge tightly around 7–9%, demonstrating that 171 evaluable tests provide sufficient data to dominate the likelihood regardless of the prior specification. Vertical grey dotted lines indicate clinically relevant positivity thresholds at 5% and 10%. The posterior probability that the true positivity rate exceeds 10% was below 25% under all three priors; the probability of exceeding 20% was negligible across all models.

PJP, *Pneumocystis jirovecii* pneumonia; PCR, polymerase chain reaction; CMI, Clinical Microbiology and Infection; CI, credible interval; θ, true institutional positivity rate.

**Supplementary Figure 2. Predictors of PJP in immunocompromised children.**

Forest plot displaying univariable (blue circles) and multivariable (red triangles) odds ratios with 95% confidence intervals from Firth penalized logistic regression at the test level (n=171; positive PCR=13). Predictors are displayed on a log10 scale. BDG: 1,3-β-D-glucan; LDH: lactate dehydrogenase; PJP: *Pneumocystis jirovecii* pneumonia; PCR: polymerase chain reaction.

**Supplementary Figure 3. Overall survival in immunocompromised children undergoing *Pneumocystis jirovecii* PCR testing.**

Kaplan-Meier survival curves with 95% confidence intervals (shaded) from the date of the index PCR test. Panel A shows all evaluable pediatric patients (n=104; events=14), with survival of 97%, 96%, and 92% at 90, 180, and 365 days respectively. Panel B shows PJP patients only (n=10; events=2), with survival of 100% at 90 and 180 days, declining to 90% at 365 days. Censored observations are marked with +. Two patients were excluded from survival analysis due to incomplete follow-up documentation. PJP: *Pneumocystis jirovecii* pneumonia; PCR: polymerase chain reaction.

**Supplementary Figure 4: Testing Volume in Immunocompromised Children Over Time, 2015–2023.** Stacked bar chart showing the annual number of *Pneumocystis jirovecii* pneumonia (PJP) PCR tests performed in pediatric patients (age ≤18 years) at LKH University Hospital Graz between 2015 and 2023. Bars are stratified by PCR result: positive (red), negative (blue), and not evaluable (grey). Annotations above each bar indicate the number of positive tests relative to the total number of tests performed that year (positive/total). The study period encompasses 171 evaluable tests from 106 unique patients. Annual positivity rates remained low and stable throughout the observation period despite a progressive increase in overall testing volume, most pronounced between 2018 and 2021, consistent with broadening clinical indications for PJP PCR testing in non-HIV immunocompromised populations. Two thousand twenty-two and 2023 represent partial or lower-volume years; individual date entries with implausible year values were corrected to the most plausible calendar year based on clinical record review prior to aggregation.

| **Predictor** | **OR (95% CI)** | **p-value** | **OR (95% CI)** | **p-value** |
| --- | --- | --- | --- | --- |
|  | ***Primary analysis (n=171, events=13)*** |  | ***Sensitivity analysis (n=106, events=10)*** |  |
| BDG (per 100 pg/mL) | 1.37 (1.14-1.73) | 0.001 | 1.84 (1.34-3.42) | <0.001 |
| LDH (per 100 U/L) | 1.51 (1.09-2.10) | 0.014 | 1.17 (0.66-1.86) | 0.545 |
| PJP prophylaxis (Yes vs No) | 0.60 (0.15-2.29) | 0.456 | 0.58 (0.07-4.18) | 0.563 |
| Bilateral infiltrates (Yes vs No) | 1.85 (0.37-13.96) | 0.482 | 1.01 (0.13-14.32) | 0.994 |

**Supplementary Table 1: Sensitivity Analysis Multivariable Firth Penalized Logistic Regression Restricted to the Index Test per Patient.**

Comparison of multivariable Firth penalized logistic regression results between the primary test-level analysis and a pre-specified sensitivity analysis restricted to the index test which was PJP-PCR in all cases per patient. The primary analysis included all 171 evaluable tests from the full study cohort, of whom 13 had a positive PCR result. The sensitivity analysis was restricted to the earliest evaluable test per individual, yielding 106 unique patients with 10 positive PCR results, thereby eliminating within-patient correlation arising from repeated testing.

All four pre-specified predictors were retained in both models: serum 1,3-β-D-glucan (BDG, scaled per 100 pg/mL), lactate dehydrogenase (LDH, scaled per 100 U/L), PJP prophylaxis status at the time of testing (Yes vs No), and bilateral pulmonary infiltrates on chest imaging (Yes vs No). Firth penalized logistic regression was used throughout to address small-sample bias and the risk of complete separation associated with low event rates. Odds ratios (OR) with 95% profile penalized likelihood confidence intervals (CI) and two-sided p-values are reported for each predictor in each model.

Rows with light red shading indicate predictors reaching nominal statistical significance (p<0.05) in the primary analysis. The overall model fit was statistically significant in the primary analysis (likelihood ratio test p=9.5×10⁻⁷ on 4 degrees of freedom). BDG remained the most robust predictor across both models, with the point estimate strengthening in the sensitivity analysis (OR 1.37 primary vs OR 1.84 sensitivity), supporting its stability independent of repeated-measurement structure. The attenuation of the LDH association in the sensitivity analysis (OR 1.51, p=0.014 vs OR 1.17, p=0.545) suggests that this association may in part reflect within-patient laboratory trends across serial tests rather than a test-independent predictor of positivity. Prophylaxis and bilateral infiltrates were non-significant in both analyses; wide confidence intervals reflect the limited number of positive events and should not be interpreted as evidence of no effect.

OR, odds ratio; CI, 95% confidence interval; BDG, beta-D-glucan; LDH, lactate dehydrogenase; PJP, *Pneumocystis jirovecii* pneumonia; PCR, polymerase chain reaction

| **Predictor** | **n (complete)** | **OR (95% CI)** | **p-value** |
| --- | --- | --- | --- |
| Sex (Female vs Male) | 171 | 2.09 (0.70-6.82) | 0.19 |
| Age (per 1 year) | 171 | 1.00 (1.00-1.00) | 0.30 |
| Prior SCT (Yes vs No) | 167 | 0.73 (0.14-2.64) | 0.66 |
| PJP prophylaxis (Yes vs No) | 171 | 0.44 (0.12-1.35) | 0.15 |
| Leukocytes (per 1 G/L) | 171 | 1.03 (0.97-1.09) | 0.29 |
| Absolute neutrophil count (per 1 G/L) | 171 | 1.02 (0.92-1.10) | 0.65 |
| Absolute lymphocyte count (per 1 G/L) | 171 | 0.15 (0.03–0.35) | **<0.001** |
| Hemoglobin (per 1 g/dL) | 171 | 0.91 (0.66-1.21) | 0.52 |
| Platelets (per 50 G/L) | 171 | 1.06 (0.90-1.24) | 0.44 |
| CRP (per 100 mg/L) | 171 | 2.35 (1.36-4.41) | **0.003** |
| LDH (per 100 U/L) | 171 | 1.78 (1.38-2.37) | **<0.001** |
| Bilirubin (per 1 mg/dL) | 171 | 1.79 (1.04-3.52) | **0.04** |
| Creatinine (per 1 mg/dL) | 171 | 1.05 (0.41-1.42) | 0.81 |
| Beta-D-Glucan (per 100 pg/mL) | 171 | 1.53 (1.28-1.89) | **<0.001** |

**Supplementary Table 2: Exploratory Univariable Firth Penalized Logistic Regression All Predictors of PJP (Test-Level).**

Univariable Firth penalized logistic regression was performed for 15 clinical and laboratory predictors of PJP at test level (n = 171 evaluable tests; 13 PCR-positive events). Firth penalization was applied throughout to address small-sample bias and the risk of complete separation associated with the low event rate. Each predictor was modelled independently without adjustment for other variables.

Continuous predictors were scaled to clinically interpretable units to avoid odds ratios indistinguishable from unity on a per-unit basis: leukocytes and absolute neutrophil count per 1 G/L; hemoglobin per 1 g/dL; platelets per 50 G/L; C-reactive protein per 100 mg/L; lactate dehydrogenase per 100 U/L; bilirubin and creatinine per 1 mg/dL; and beta-D-glucan per 100 pg/mL. Binary and categorical predictors are reported as the non-reference category versus the reference category as indicated. Missing values were imputed using multiple imputation by chained equations (MICE; m = 20, predictive mean matching); three predictors had incomplete data (prior SCT: n = 167 complete; concurrent viral detection: n = 168 complete) and all remaining predictors were fully observed (n = 171).

Odds ratios (OR) with 95% profile penalized likelihood confidence intervals (CI) and two-sided p-values are reported. No correction for multiple comparisons was applied; all findings are explicitly exploratory and hypothesis-generating given the limited number of positive events. Rows with light red shading indicate nominal statistical significance (p < 0.05, unadjusted); these should not be interpreted as confirmed associations without independent validation. Three predictors reached nominal significance: C-reactive protein (OR 2.35 per 100 mg/L, p = 0.003), lactate dehydrogenase (OR 1.78 per 100 U/L, p < 0.001), and beta-D-glucan (OR 1.53 per 100 pg/mL, p < 0.001). Bilirubin showed a borderline association (OR 1.79 per 1 mg/dL, p = 0.04) that would not survive Bonferroni correction for 15 comparisons (corrected threshold p < 0.0033 [0.05/15]). ALC also reached corrected significance (p<0.001), the fourth predictor to do so alongside CRP, LDH, and BDG.

OR, odds ratio; CI, 95% confidence interval; SCT, stem cell transplantation; PJP, *Pneumocystis jirovecii* pneumonia; PCR, polymerase chain reaction; CRP, C-reactive protein; LDH, lactate dehydrogenase; BDG, 1,3-β-D-glucan; MICE, multiple imputation by chained equations.

| **Metric** | **Test level, 33 pg/mL** | **Test level, ≥80 pg/mL** | **Patient level, 33 pg/mL** | **Patient level, ≥80 pg/mL** |
| --- | --- | --- | --- | --- |
| Sensitivity | 84.6% (57.8–95.7) | 84.6% (57.8–95.7) | 90.0% (59.6–98.2) | 90.0% (59.6–98.2) |
| Specificity | 89.2% (83.4–93.2) | 96.8% (92.8–98.6) | 88.5% (80.6–93.5) | 96.9% (91.2–98.9) |
| PPV | 39.3% (23.6–57.6) | 68.8% (44.4–85.8) | 45.0% (25.8–65.8) | 75.0% (46.8–91.1) |
| NPV | 98.6% (95.0–99.6) | 98.7% (95.4–99.6) | 98.8% (93.7–99.8) | 98.9% (94.2–99.8) |
| AUC | 0.835 (0.65–1.00) | — | 0.829 (0.64–0.99) | — |

**Supplementary Table 3. Diagnostic performance of serum 1,3-β-D-glucan for *Pneumocystis jirovecii* pneumonia at the exploratory (33 pg/mL) and manufacturer-recommended (≥80 pg/mL) cut-offs.**

Performance is reported at the test level (n = 171 evaluable tests; 13 PCR-positive) and at the patient level (n = 106 unique patients; 10 PCR-positive), the latter restricted to the index test per patient to remove within-patient correlation from repeated testing. Sensitivity, specificity, positive predictive value (PPV), and negative predictive value (NPV) are shown for each cut-off with exact (Wilson) 95% confidence intervals; predictive values are reported at the prevalence observed in this cohort and are therefore not generalizable to populations with a different pre-test probability. The area under the curve (AUC) is derived from 1,3-β-D-glucan as a continuous predictor and is independent of any cut-off; a single value is therefore reported for each analysis level. The reference standard was a positive *P. jirovecii* PCR on bronchoalveolar lavage; 1,3-β-D-glucan was not used in case ascertainment. AUC, area under the receiver operating characteristic curve; BDG, 1,3-β-D-glucan; CI, confidence interval; NPV, negative predictive value; PJP, *Pneumocystis jirovecii* pneumonia; PCR, polymerase chain reaction; PPV, positive predictive value.
